# Supplementary material for: The periodical cicada four-year acceleration hypothesis revisited and the polyphyletic nature of Brood V, including an updated crowd-source enhanced map (Hemiptera: Cicadidae: Magicicada)
Source: PeerJ. 2018 Jul 31;6:e5282. doi: 10.7717/peerj.5282 (PMC6074776; doi:10.7717/peerj.5282)
Supplement: Supplemental Information 1 [file peerj-06-5282-s001.docx]

# Supplemental Information

- *Carbon Advocate* (Leighton PA), March 6, 1880. Column 1. “Locusts are to visit us next summer, in accordance with long established customs, in swarms.”
- *Carbon Advocate* (Leighton PA), May 29, 1880 Column 6. “Locusts are again heard in the woods ‘singing so sweetly in the boughs of the old oak trees.’ These winged creatures are more numerous than ever this year.”
- *Allentown Morning Call*, June 15, 1897, Page 1. Local report. “Seventeen-Year Locusts Here Again. The seventeen-year locusts have appeared in many portions of Schuylkill county.”
- *Allentown Morning Call*, June 23, 1931, Page 8. Local. “17-Year Locusts Will Soon Go Into Action. Among other insect visitors this month the ’17-year’ locus will come to certain parts of PA. The adults of Brood V of the Periodical Cicada, as the entomologists call the locus, are due to appear. H.N. Worthley, research entomologist at the PA State College, reports that, in previous appearances, this brood has been found in two well-defined areas: Schuylkill, Carbon and Berks counties in the eastern part of the state, and Allegheny, Westmoreland, Washington, Greene and Payette in the western part.”
- *Allentown Morning Call*, June 13, 1931, Page 3. Local Mauch Chunk report. “Locusts have made their appearance in lower Carbon county and are reported to be doing damage to trees. So far none of the locusts have been seen hereabout.”
- *Allentown Morning Call,* June 29, 1931, Page 12. Local Leighton report. “Locusts are reported to have made their appearance in the lower end of the county but their song has not been heard in the upper part as well. They usually visit the farming sections and woodland.”
- *Allentown Morning Call*, July 5, 1931, Page 8. Local report. “Locusts are reported swarming in various counties but so far none have arrived here.”
- *Allentown Morning Call*, June 16, 1948, Page 25. Local “Chatter” section. “Locusts in large swarms sighted on East Mauch Chunk Mountain, Hacklebirnie area and eastern end of Nesquehoning.”
- *Allentown Morning Call*, May 17, 1965, Page 2. Local short paragraph in the “Newsy & Short” section. “A new teen-age singing group is coming to PA for a stand of 5 to 6 weeks … this group is not the Rolling Stones or the Dave Clark Five. It is closer to the Beatles. It is a brood of insects – periodical cicadas, more commonly known as 17 year locusts.”
- *Allentown Morning Call*, June 20, 1965, Page 74. Local “Garden Tips” section. “17-Year Locusts. Some areas of PA will be having problems with the 17 year locusts. Carbon County had its invasion. According to the experts Lehigh County should not have real damage done because of our locations. The locusts or cicada batch out in broods and Lehigh County is not scheduled for 1965.”
- *Allentown Morning Call*, June 6, 1965. Page 22. Local article. “17-Year Locust Returns. A new brood of the periodical cicada or 17-year locust has emerged from the ground in parts of Carbon County … Known as the Brood V, the locust population emerged from the ground about two weeks ago in Carbon County…”
- *Allentown Morning Call*, May 17, 1999, page 20. AP story “After 17 years, periodical cicadas return.” Mentions eastern Ohio, most of West Virginia and small parts of PA and VA. But no local mention.
- *Allentown Morning Call*, June 6, 1999, Page 41. Local story about Hickory Run park and campground mentions “The few visitors able to linger beyond Sunday night will enjoy the seclusion when the only sounds may be that of a pileated woodpecker practicing its drumroll on a hollow tree or the night-sounds of crickets and cicadas.” Nothing specific though.
- *Allentown Morning Call*, July 12, 1999, Page 11. Local story on Screech owls. “In the warmer months, insects, earthworms, crayfish and other invertebrates supplement their diet. In the last month local screech owls also caught cicadas in flight, removing their wings and swallowing the insects in one gulp.”
